# Serine‐227 in the N‐terminal kinase domain of RSK2 is a potential therapeutic target for mantle cell lymphoma

**DOI:** 10.1002/cam4.3136

**Published:** 2020-05-18

**Authors:** Yayoi Matsumura‐Kimoto, Taku Tsukamoto, Yuji Shimura, Yoshiaki Chinen, Kazuna Tanba, Saeko Kuwahara‐Ota, Yuto Fujibayashi, Daichi Nishiyama, Reiko Isa, Junko Yamaguchi, Yuka Kawaji‐Kanayama, Tsutomu Kobayashi, Shigeo Horiike, Masafumi Taniwaki, Junya Kuroda

**Affiliations:** ^1^ Division of Hematology and Oncology Department of Medicine Kyoto Prefectural University of Medicine Kyoto Japan; ^2^ Center for Molecular Diagnostics and Therapeutics Kyoto Prefectural University of Medicine Kyoto Japan

**Keywords:** B cell tumorigenesis, mantle cell lymphoma, molecular target, RSK2

## Abstract

RSK2 is a serine/threonine kinase downstream signaling mediator in the RAS/ERK signaling pathway and may be a therapeutic target in mantle cell lymphoma (MCL), an almost incurable disease subtype of non‐Hodgkin lymphoma. In this study, serine‐227 (RSK2^Ser227^) in the N‐terminal kinase domain (NTKD) of RSK2 was found to be ubiquitously active in five MCL‐derived cell lines and in tumor tissues derived from five MCL patients. BI‐D1870, an inhibitor specific to RSK2‐NTKD, caused RSK2^Ser227^ dephosphorylation, and thereby, induced dose‐dependent growth inhibition via G_2_/M cell cycle blockade and apoptosis in four of the five cell lines, while one cell line showed only modest sensitivity. In addition, RSK2 gene knockdown caused growth inhibition in the four BI‐D1870‐sensitive cell lines. Comparative gene expression profiling of the MCL‐derived cell lines showed that inhibition of RSK2^Ser227^ by BI‐D1870 caused downregulation of oncogenes, such as c‐MYC and MYB; anti‐apoptosis genes, such as BCL2 and BCL2L1; genes for B cell development, including IKZF1, IKZF3, and PAX5; and genes constituting the B cell receptor signaling pathway, such as CD19, CD79B, and BLNK. These findings show that targeting of RSK2^Ser227^ enables concomitant blockade of pathways that are critically important in B cell tumorigenesis. In addition, we found favorable combinatory growth inhibitory effects of BI‐D1870 with inhibitors of BTK (ibrutinib), AKT (ipatasertib), and BCL2 (venetoclax) in cell characteristic‐dependent manners. These results provide a rationale for RSK2^Ser227^ in the NTKD as a potential therapeutic target in MCL and for future development of a novel bioavailable RSK2 NTKD‐specific inhibitor.

## Graphical abstract

RSK2Ser227 in the N‐terminal kinase domain (NTKD) of RSK2 was found to be ubiquitously active in mantle cell lymphoma (MCL). RSK2 concomitantly regulates series of genes involved in oncogenesis, apoptosis, B cell development, and B cell receptor signaling. RSK2Ser227 in the NTKD is a potential therapeutic target in MCL.

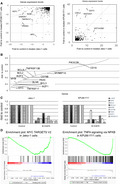

## 1 INTRODUCTION

Mantle cell lymphoma (MCL) is a relatively rare, but aggressive, disease subtype of non‐Hodgkin lymphoma. MCL is characterized by reciprocal chromosomal translocation of the immunoglobulin heavy chain (IgH) at chromosome 14q32 and cyclin D1 at chromosome 11q13, resulting in overexpression of cyclin D1 (CCND1) and cyclin‐dependent kinase (CDK) activation.^1^ MCL is almost incurable, despite the advent of rituximab‐containing immunochemotherapy and molecular‐targeted therapeutics such as ibrutinib, a BTK inhibitor; bortezomib, a proteasome inhibitor; lenalidomide, an immunomodulatory drug; copanlisib, a PI3K inhibitor, and sirolimus, a mTOR inhibitor.^2^, ^3^, ^4^, ^5^, ^6^, ^7^, ^8^ Therefore, identification of a novel molecular target is an unmet need for development of a more effective therapeutic agent for MCL.

The RAS/RAF/MEK/ERK signaling pathway promotes cell survival, cell cycle progression for cell proliferation, and cell migration of various types of cells, including hematopoietic cells, under physiologic conditions. This signaling pathway is frequently abnormally activated by extrinsic stimuli such as soluble factors and cell adhesion, and also by various intrinsic cytogenetic or molecular abnormalities, such as activating mutations of EGFR, RAS, or RAF, or chromosomal abnormalities. These collectively lead to constitutive activation of the signaling pathway, which then plays a pivotal role in cancer development and progression. The RAS/RAF/MEK/ERK pathway includes attractive anticancer therapeutic target molecules, such as MEK, which is targeted by trametinib, and RAF, targeted by vemurafenib.^9^, ^10^

RSK2 (ribosomal protein S6 kinase, 90‐KD, 3; RPS6KA3) is a serine/threonine kinase that has critical functional roles as a downstream signaling mediator of the RAS/RAF/MEK/ERK signaling pathway. RSK2 was first identified in the 1990’s and has been linked to physiologic processes in metabolism, cardiac systems, and neurology,^11^, ^12^, ^13^ and to onset and progression of various cancers.^14^, ^15^, ^16^, ^17^ Similarly to other RSK family proteins, RSK2 possesses two functional domains: an N‐terminal kinase domain (NTKD) and a C‐terminal kinase domain (CTKD).^18^, ^19^ Under physiologic conditions, ERK phosphorylates Thr577 in the CTKD, and this leads to subsequent activation of Thr365/Ser369 in the linker domain of RSK2. Phosphorylation of this linker domain then leads to docking of 3‐phosphoinositide‐dependent protein kinase 1 (PDPK1) to allow phosphorylation of Ser227 (RSK2^Ser227^) in the NTKD. The RSK2‐NTKD serves as the output module for the RAS/ERK signal by phosphorylating a series of substrates, including molecules associated with cell proliferation and survival.^20^

We have shown that PDPK1 is constitutively expressed and hyperactivated by autophosphorylation, and constitutively activates RSK2^Ser227^ in the NTKD regardless of the activation status or molecular abnormalities upstream in the RAS/RAF/MEK/ERK pathway. These events are critical in the pathophysiology of multiple myeloma (MM) and MCL.^21^, ^22^, ^23^, ^24^ While RSK2 and AKT are the two major substrates of PDPK1, we have shown that RSK2, not AKT, plays a central role as the downstream effector of PDPK1 in MM and MCL.^22^, ^24^ However, the exact biological and molecular functions of RSK2 in MCL remain to be determined. In this study, we investigated the activation status of RSK2^Ser227^ in MCL and the functional roles of RSK2, and especially RSK2^Ser227^, in MCL‐derived cell lines to examine the value of RSK2 as a therapeutic target in MCL.

## 2 METHODS

### 2.1 Patient samples and immunochemical (IHC) staining of phosphorylated (p)‐RSK^Ser227^

Biopsied tumor specimens and medical records for five patients with MCL (Table S1) were obtained with informed consent in accordance with the Declaration of Helsinki. Tumor samples were obtained from the spleen in one case, tonsil in one case, and lymph nodes in three cases. Formalin‐fixed and paraffin‐embedded tissue samples were subjected to IHC staining by anti‐p‐RSK2^Ser227^ monoclonal antibody (Bioss) or anti‐CCND1 antibody (Santa Cruz Biotechnology), as previously described.^21^, ^22^, ^23^, ^24^, ^25^ These analyses were approved by the Institutional Review Board (RBMR‐G‐124‐9).

### 2.2 Cells and reagents

Human MCL cell lines (MINO, Z‐138, Jeko‐1, and JVM‐2) were obtained from American Type Culture Collection and KPUM‐YY1 cells previously established in our laboratory^25^ were used in the study. MINO cells and Jeko‐1 cells have been reported to harbor TP53 gene mutation, while Jeko‐1 cells and JVM‐2 cells possess MLL2 gene mutation. Neither ATM gene mutation nor CCND1 gene mutation has been detected in MINO, Z‐138, Jeko‐1, or JVM‐2 cells.^26^, ^27^, ^28^, ^29^ Direct sequence for mutational hotspot region between exons 4 and 9 of TP53 gene identified an intronic base substitution of c.375 + 1G>T at downstream of exon 4 which has been predicted to be pathogenic in COSMIC (Catalogue Of Somatic Mutations In Cancer) v90 Database (https://cancer.sanger.ac.uk/cosmic/mutation/overview?id=87919856), but showed no mutation in the corresponding exons in KPUM‐YY1 (data not shown). The combination of G‐banding and Spectral karyotyping was performed as described previously,^25^ and the karyotype was described in accordance with the International System for Human Cytogenetic Nomenclature ISCN 2013.^30^ In brief, all five cell lines utilized in this study showed complex chromosomal abnormalities, including t(11;14)(q13;q32). The 8q24 rearrangement involving *MYC* locus was observed in all but JVM‐2 cells (Table S2). Peripheral blood mononuclear lymphocytes were isolated from peripheral blood of five healthy volunteers by density gradient centrifugation using Ficoll**‐**Paque PLUS (GE Healthcare Life Science). Cells were maintained in RPMI‐1640 (Wako) containing 10% fetal calf serum (Life Technologies), 2 mmol/L L‐glutamate, and penicillin/streptomycin at 37°C in humidified 95% air and 5% CO_2_. BI‐D1870, an RSK2 NTKD‐specific inhibitor, and venetoclax (ABT‐199), a BCL‐2 inhibitor (both Cayman Chemical Company), FMK, an inhibitor of the RSK2 CTKD (Axson Medchem), and ibrutinib and ipatasertib (GDC‐0068), the AKT inhibitors (Selleck Biotech Ltd.) were used in the study.^18^, ^19^, ^31^, ^32^

### 2.3 RNA interference

RNA interference (RNAi) targeted against RSK2 was performed by transfecting small‐interfering RNA (siRNA) into Jeko‐1 and KPUM‐YY1 cells, using Hemagglutinating Virus of Japan‐envelope vector (Ishihara Sangyo Kaisha, Ltd., Osaka, Japan). The sequences of the sense strands of the two siRNAs used against RSK2 were 5′‐UGG CUC CAG AAG UAG UUA ATT‐3′ (Si‐#1) and 5′‐GGC CUG AAG AUA CAU UCU A‐3′ (Si‐#2), and those of the antisense siRNAs were 5′‐UUA ACU ACU UCU GGA GCC ATT‐3′ for Si‐#1 and 5′‐UAG AAU GUA UCU UCA GGC C‐3′ for Si‐#2. The sequences for controls for Si‐#1 and Si‐#2 were 5′‐UCU UAA UCG CGU AUA AGG CTT‐3′ and 5′‐GCC UAU ACG CGA UUA AGA TT‐3′, respectively.

### 2.4 Quantitative reverse transcription‐polymerase chain reaction (RT‐PCR)

Total RNA was extracted using a RNeasy Mini Kit (Qiagen). Primers were purchased from Hokkaido Systems Science Co., Ltd. (Table S3). Quantitative RT‐PCR was performed using Fast SYBR Green Master Mix with a StepOne Plus instrument (Applied Biosystems). Transcriptional levels of target genes were adjusted based on that of β‐actin using Taqman β‐actin (ACTB) control reagents. Three independent experiments were performed for each target gene, and results are expressed as the mean* ± *standard deviation.

### 2.5 Assays for growth inhibition and apoptosis

Cells were seeded at 2.5 × 10^5^ cells/mL and treated with various concentrations of BI‐D1870, FMK, ibrutinib, venetoclax, or ipatasertib for 48 hours. Growth inhibition was analyzed by a modified MTT [3‐(4,5‐dimethylthiazol‐2‐yl)‐2,5‐diphenyltetrazolium bromide] assay using a Cell Counting Kit‐8 (Dojindo Molecular Technologies). For analysis of apoptosis, cells were counterstained with Annexin V‐fluorescein isothiocyanate and propidium iodide (PI) and subjected to flow cytometric analysis. For evaluation of the cell cycle distribution based on cellular DNA content, cells were fixed with ice‐cold 70% ethanol, stained with PI, and then analyzed by flow cytometry. Data were analyzed using FlowJo software ver. X (Tomy Digital Biotechnology).

### 2.6 Western blot analysis

Western blot analysis was conducted as described previously,^21^, ^22^, ^23^, ^24^, ^25^ using primary antibodies against RSK2 (Santa Cruz Biotechnology), p‐RSK2^Ser227^, p‐RSK2^Thr529^, AKT, p‐AKT^Thr308^, ERK, p‐ERK, polo‐like kinase 1 (PLK1), p‐PLK1, Aurora kinase B (AURKB), p‐AUKRB, PDPK1 (Cell Signaling Technology), and β‐actin (ACTB) (Sigma‐Aldrich).

### 2.7 Microarray analysis, signal pathway analysis, and gene set enrichment analysis (GSEA)

Jeko‐1 and KPUM‐YY1 cells were treated with BI‐D1870 at IC_80_ for each cell line for 6 hours. Total RNA was isolated using a RNeasy Mini Kit, and the gene expression profile (GEP) was analyzed with a Clariom S array (Affymetrix), a GeneChip WT Plus Reagent Kit (Thermo Fisher Scientific) and a GeneChip Scanner 7G (Affymetrix). Data were analyzed using Affymetrix Transcriptome Analysis Console (TAC) software ver. 4.0.0.25.and performed robust multiarray (RMA) normalization with default parameters using TAC programming. Genes with at least a 2.0‐ or 0.5‐fold difference in the expression level from those in untreated control cells were considered to be significant. Gene expression changes of several representative genes detected by GEP were validated by the quantitative RT‐PCR described above. Signal pathway analysis was performed using Ingenuity Pathway Analysis (IPA) software (Ingenuity Systems). GSEA was performed using the software GSEA (https://www.gsea‐msigdb.org/gsea/index.jsp).^33^

### 2.8 Drug combination assays

Combinatory growth inhibitory effects of BI‐D1870 with ibrutinib, venetoclax, or ipatasertib were examined in Jeko‐1, KPUM‐YY1, MINO, and Z‐138 cells. In KPUM‐YY1 cells, the combinatory growth inhibitory effects of BI‐D1870 and ibrutinib was evaluated using CalcuSyn software (Biosoft). Briefly, cells were treated with eight concentrations (0.25, 0.5, 0.75 1.0, 1.25, 1.5, 1.75, 2.0 × IC_50_) of either BI‐D1870, or ibrutinib, or by both agents for 48 hours, and were subjected to a modified MTT assay. Fractional effect concentrations (ie, a fractional effect of 0.25 equals a 25% growth inhibitory effect) and the combination index (CI) were calculated with CalcuSyn (Biosoft). This method facilitates quantification of synergism (CI < 1) and antagonism (CI > 1) at different doses and effect levels. CI calculations were conducted on the assumption that drug mechanisms were not mutually exclusive. Combinatory effect of BI‐D1870 and ibrutinib was not examined in other cell lines without sensitivity to ibrutinib with its clinically achievable concentration. Since many of cell lines examined showed modest sensitivity to venetoclax and ipatasertib, it was not possible to perform conventional combinatory assays with those and BI‐D1870, such as that analyzed by CalcuSyn or an assay using isobologram analysis. Therefore, we combined BI‐D1870 with venetoclax or ipatasertib at concentrations at which the agents showed modest antiproliferative effects (20%‐40% growth inhibition) in the four BI‐D1870‐sensitive MCL‐derived cell lines. Cells were treated with BI‐D1870 or partner agent alone, or in combination for 48 hours and evaluated using the modified MTT assay.

## 3 RESULTS

### 3.1 RSK2^Ser227^ is phosphorylated in patient‐derived MCL cells and MCL‐derived cell lines

**FIGURE 1:**
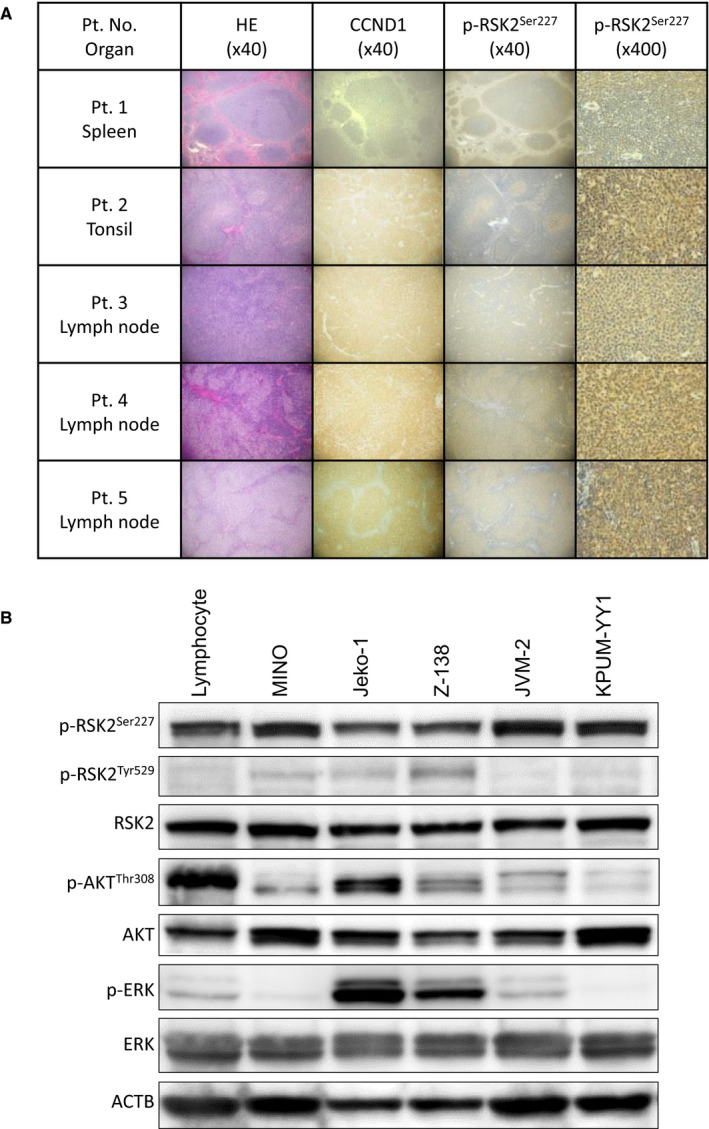
Phosphorylation status of RSK2^Ser227^ in patient (Pt.)‐derived mantle cell lymphoma (MCL) tissues and MCL‐derived cell lines. A, Hematoxylin‐eosin (HE) and immunohistochemical staining for cyclin D1 (CCND1) and phosphorylated (p)‐RSK2^Ser227^ in tumor biopsy specimens from patients (Pt. Nos. 1‐5) with MCL. Cytoplasms of lymphoma cells were positive for CCND1 and p‐RSK2^Ser227^. B, Expression patterns of total RSK2, p‐RSK2^Ser227^, p‐RSK2^Tyr529^, ERK, p‐ERK, AKT, and p‐AKT^Thr308^ examined by Western blotting in five MCL‐derived cell lines and normal lymphocytes. β‐Actin (ACTB) was used as the internal control. RSK2^Ser227^ was phosphorylated regardless of the activation status of RSK2^Tyr529^, ERK, and AKT in all five cell lines

The phosphorylation status of RSK2^Ser227^ in patient‐derived tumor tissues from five MCL patients (Table S1) was evaluated by IHC staining. Tumor cells in the MCL lesions were positive for p‐RSK2^Ser227^ in all five patients (Figure 1A). We then investigated the phosphorylation status of RSK2^Ser227^ and its relationship with those of ERK and RSK2^Tyr529^ in the RSK2‐CKTD, which are upstream of RSK2^Ser227^; and that of AKT^Thr308^, which is also a substrate of PDPK1, in MINO, Jeko‐1, Z‐138, JVM‐2, and KPUM‐YY1 cells. Phosphorylation of AKT and ERK differed among the cell lines, and RSK2^Tyr529^ was not phosphorylated or was only modestly phosphorylated in the five cell lines. In contrast, RSK2^Ser227^ was phosphorylated in all five cell lines (Figure 1B). These results indicate that activation of ERK and RSK2^Tyr529^ is not always a prerequisite for phosphorylation of RSK2^Ser227^ in MCL cells. Furthermore, while RSK2^Ser227^ and AKT^Thr308^ share PDPK1 as a common upstream kinase, our results indicate that, in contrast to RSK2^Ser227^, AKT activation is not consistent in all MCL‐derived cell lines.

### 3.2 In vitro growth inhibitory effects by inhibition of RSK2 in MCL‐derived cell lines

**FIGURE 2:**
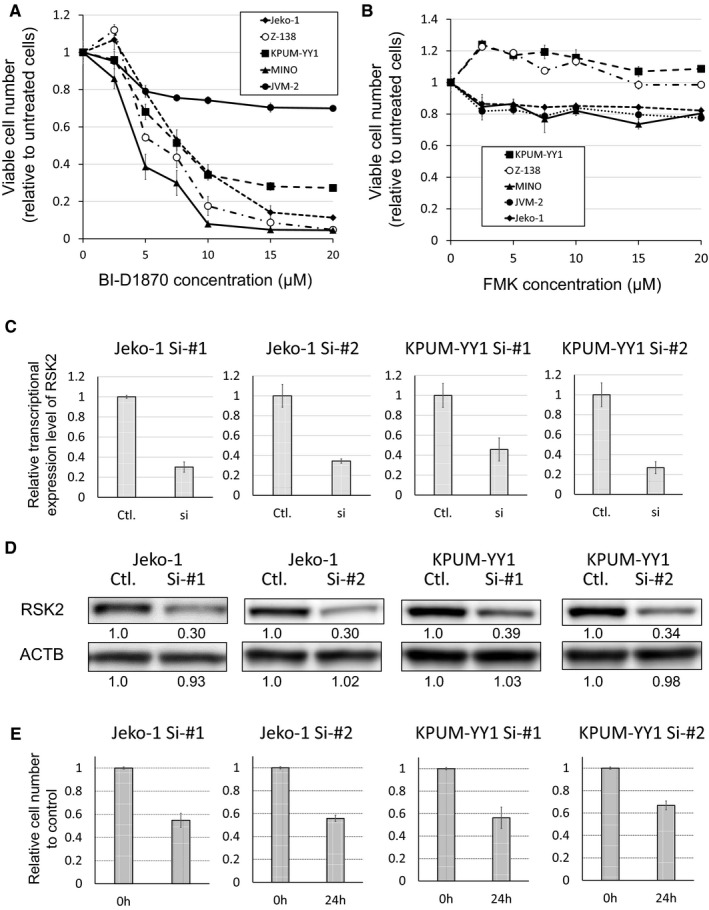
Growth inhibitory effects by inhibition of RSK2 in MCL‐derived cell lines. A,B, Growth inhibitory effects of BI‐D1870, an inhibitor of RSK2^Ser227^ in the NTKD (A), and FMK, an inhibitor of RSK2‐CTKD (B), on five MCL cell lines. Cells were exposed to various concentrations of BI‐D1870 or FMK for 48 h, and the relative numbers of viable cells were measured by modified MTT assay. IC_50_ values for the four BI‐D1870‐sensitive cell lines Jeko‐1, KPUM‐YY1, MINO, and Z‐138 were 8.9, 9.5, 5.2, and 7.4 µmol/L, respectively. Data are shown as mean ± SD of three independent experiments. C,D, RSK2 gene knockdown by RNA interference (RNAi) in Jeko‐1 and KPUM‐YY1 cells. Expression levels of RSK2 in RSK2‐knockdown cells (si) relative to those transfected with control siRNAs (Ctl.) are shown as the mean ± SD of three independent experiments (C). Protein levels were measured using Image‐J software (D). E, Numbers of viable RSK2 knockdown cells after 24 h relative to those transfected with Ctl. siRNAs are shown as the mean ± SD of three independent experiments

To clarify the roles of RSK2 in the proliferation of MCL cells, we examined the effects of pharmacologic inhibition of RSK2‐NTKD by BI‐D1870 and of RSK2‐CTKD by FMK in these cells. Exposure to BI‐D1870 for 48 hours (Figure 2A) resulted in dose‐dependent growth inhibition in all five cell lines examined, with a weaker effect in JVM‐2 cells compared with the other four cell lines. In the BI‐D1870‐sensitive Jeko‐1, KPUM‐YY1, MINO, and Z‐138 cells, the IC_50_ values of 48‐hour treatment by BI‐D1870 were 8.9, 9.5, 5.2, and 7.4 µmol/L, respectively. In contrast, there was no prominent growth inhibitory effect up to 20 µmol/L FMK in all five MCL‐derived cell lines (Figure 2B).

To exclude an off‐target effect (that is, not on RSK2‐NTKD) of BI‐D1870, we examined whether BI‐D1870 had inhibitory effects on its other known targets, PLK1 and AURKB,^32^ at the IC_80_ concentration in BI‐D1870‐sensitive Jeko‐1, KPUM‐YY1, MINO, and Z‐138 cells. RSK2^Ser227^ was dephosphorylated by BI‐D1870 at IC_80_ for 6 hours in all four cell lines, whereas neither PLK1 nor AURKB was dephosphorylated by the same treatment. In contrast, the same treatment occasionally induced modest phosphorylation of PLK1 and/or AURKB in the MCL cells. AKT and ERK also showed cell context‐dependent feedback phosphorylation along with RSK2^Ser227^ dephosphorylation in MINO and Z‐138 cells treated with BI‐D1870. The PDPK1 protein level was unchanged after BI‐D1870 treatment; therefore, the mechanism underlying cellular context‐dependent AKT reactivation remains to be verified.

**FIGURE 3:**
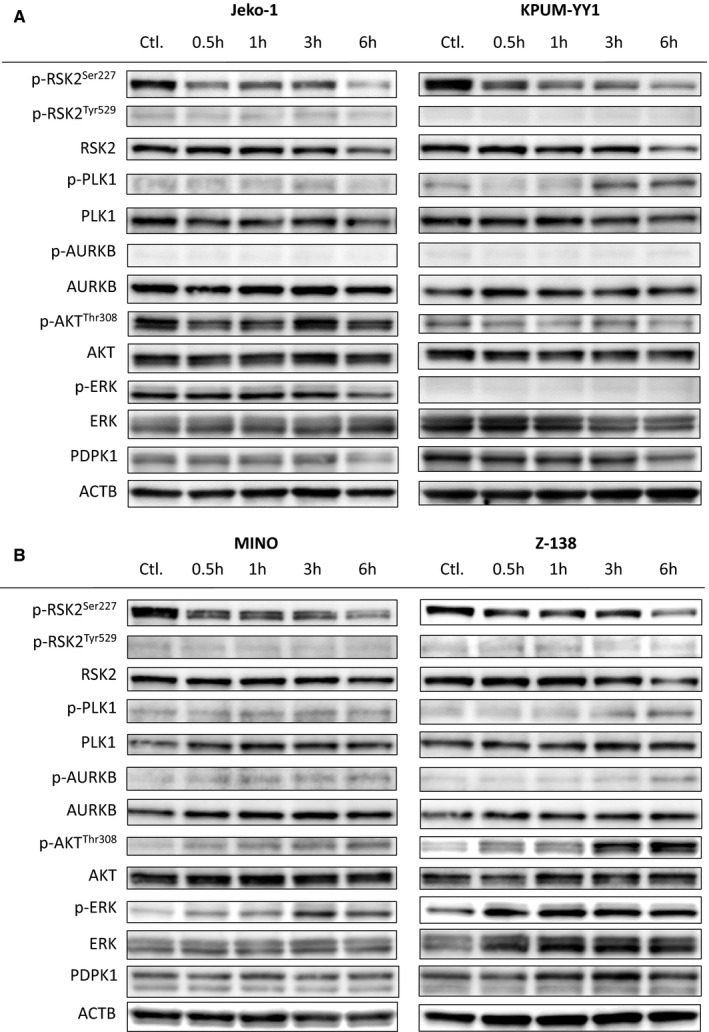
Effects of BI‐D1870 on its target protein and related kinases in four BI‐D1870‐sensitive MCL‐derived cell lines. A,B concentrations for the indicated periods. Ctl.: untreated control

These results suggest that growth inhibition by BI‐D1870 was highly likely to be directly caused by its inhibitory effect on RSK2^Ser227^ in the MCL cells (Figure 3). To verify the functional role of RSK2 further, we examined the effect of RSK2 gene knockdown in Jeko‐1 and KPUM‐YY1 cells. Partial gene knockdown of RSK2 using RNAi for 24 hours caused decreased proliferation in both cell lines (Figure 2C‐E).

### 3.3 RSK2‐NTKD inhibition induces G_2_‐M cell cycle blockade and induction of apoptosis

**FIGURE 4:**
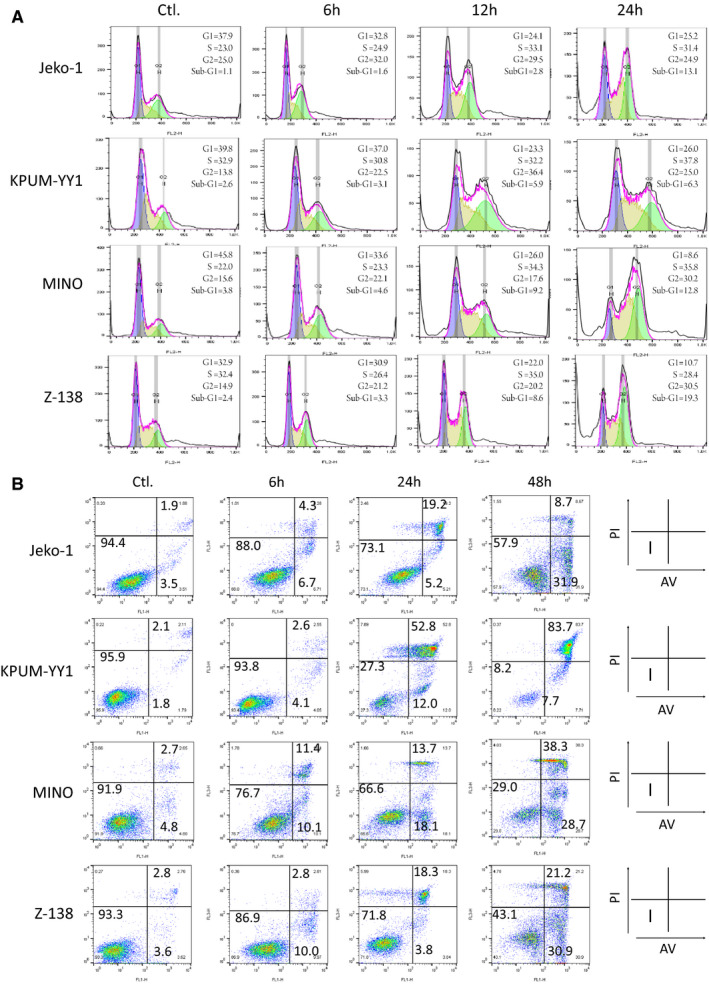
Cellular effects of treatment of BI‐D1870‐sensitive MCL‐derived cell lines with BI‐D1870. Jeko‐1, KPUM‐YY1, MINO, and Z‐138 cells were treated with BI‐D1870 at IC_80_ concentrations for the indicated periods and then subjected to (A) cell cycle analysis by measurement of DNA content, and (B) apoptosis analysis by counterstaining with Annexin‐V (AV) and propidium iodide (PI) using flow cytometry. The cell cycle distribution was calculated using FlowJo software ver. X (A). In (B), Fraction I: AV negative (−)/PI (−) viable cells; Fraction II: AV (+)/PI(−) cells undergoing early apoptosis; Fraction III: AV (+)/PI (+) cells undergoing late apoptosis. Cell ratios are shown by the numbers on fractions I, II and III

We next accessed the mechanisms of action underlying growth inhibition by RSK2 inactivation in the four BI‐D1870‐sensitive MCL‐derived cell lines. As shown in Figure 4A, treatment with BI‐D1870 at IC_80_ for up to 24 hours increased the population of G_2_/M viable cells and dying cells appeared in subG1 phases in all four cell lines. BI‐D1870 treatment generally first increased cells in the Annexin V‐positive/PI‐negative fraction (early apoptosis phase), followed by an increase of cells in the Annexin V‐positive/PI‐positive fraction (late apoptosis phase) (Figure 4B). These results collectively show that BI‐D1870 caused G_2_/M cell cycle blockade and induction of apoptosis in the MCL‐derived cell lines.

### 3.4 Molecular regulation by RSK^Ser227^ in MCL cell lines

**FIGURE 5:**
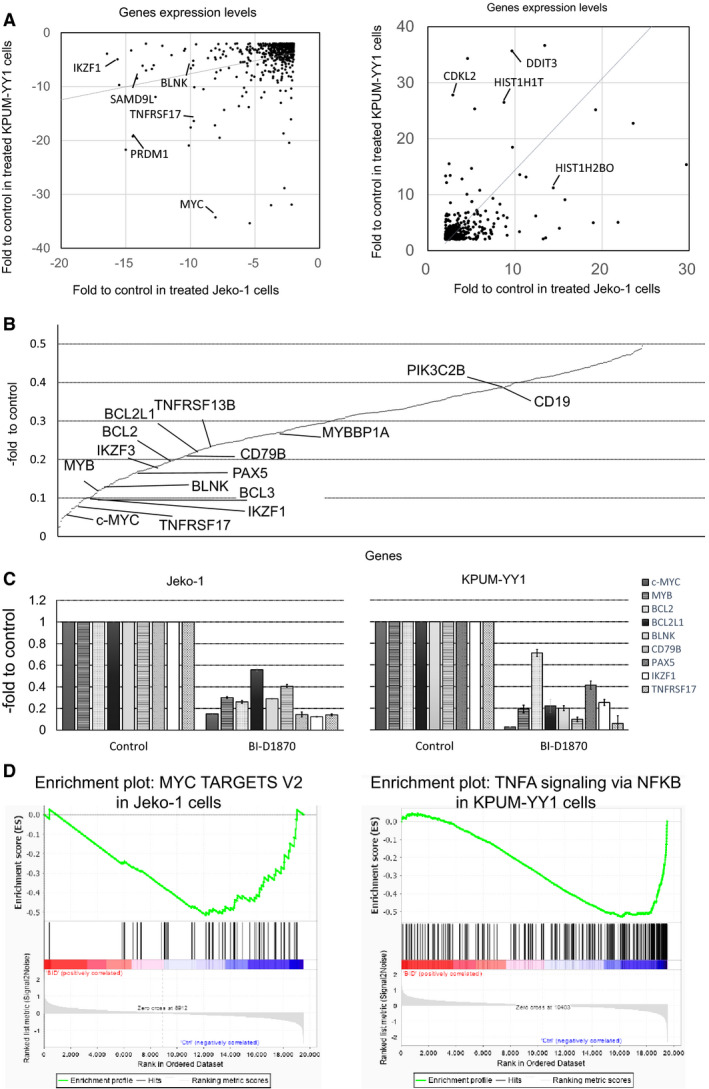
Gene expression changes induced by BI‐D1870 in MCL‐derived cell lines. A,B. KPUM‐YY1 and Jeko‐1 cells were treated with IC_80_ concentrations of BI‐D1870 for 6 h. Correlation coefficients of commonly upregulated (>2‐fold) and downregulated (<0.5‐fold) genes between Jeko‐1 and KPUM‐YY1 cells were 0.63 and 0.27, respectively (A). B, Mean expression levels of 542 commonly downregulated genes Jeko‐1 and KPUM‐YY1 cells after BI‐D1870 treatment relative to untreated cells, identified by microarray analysis. Several genes involved in lymphomagenesis, B‐cell receptor signaling, and B cell development are listed. C, Relative expression levels of c‐MYC, MYB, BCL2, BCL2L1, BLNK, CD79B, PAX5, IKZF1, and TNFRSF17 determined by quantitative RT‐PCR in two cell lines treated with BI‐D1870 at their respective IC_80_s for 6 h. Expression levels were defined as 1.0 in untreated cells. Data are shown as the mean ± SD of three independent experiments. D, Gene set enrichment plots for representative target gene sets of MYC significantly enriched in Jeko‐1 cells treated by BI‐D1870 (BID) (left) and of TNF‐α signaling via NF‐kB pathway significantly enriched in KPUM‐YY1 cells treated by BID (right) compared with untreated cells (Ctrl)

Comparative GEPs were obtained by analyzing two sets of untreated and BI‐D1870‐treated cells to identify genes commonly regulated by RSK2^Ser227^ in MCL cells. Data were deposited in National Center for Biotechnology Information‘s Gene Expression Omnibus (GSE147484). The GEPs were subjected to canonical pathway analysis with gene set classification. Jeko‐1 and KPUM‐YY1 cells were used in this analysis to avoid the influence of feedback phosphorylation of AKT or ERK after BI‐D1870 treatment, as shown in Figure 3. A >2‐fold increase or <0.5‐fold decrease of transcriptional expression level after BI‐D1870 treatment at IC_80_ for 6 hours was defined as significant. Using this definition, 1406 and 1683 genes were downregulated by BI‐D1870 in Jeko‐1 and KPUM‐YY1 cells, respectively, with 542 genes commonly downregulated in the two cell lines; and 1480 and 1132 genes were upregulated by BI‐D1870 in the respective cell lines, with 435 genes commonly upregulated (Table S4). The correlation coefficients for expression changes between Jeko‐1 and KPUM‐YY1 cells were 0.27 for the 542 downregulated genes and 0.63 for the 435 upregulated genes, suggesting that genes commonly regulated by RSK2^Ser227^ largely overlapped between Jeko‐1 and KPUM‐YY1 cells (Figure 5A). The expression changes of several genes pathophysiologically important for MCL detected in GEP analysis were confirmed by quantitative RT‐PCR (Figure 5C).

A canonical pathway analysis based on the GEPs showed that RSK2^Ser227^ inactivation by BI‐D1870 commonly caused downregulation of several genes that are critically involved in tumorigenesis, such as c‐MYC, MYB, RASGRP, and RHOH; genes related to oncogenic signaling pathways, such as the PI3K, STAT3, and NF‐kB pathways; and the anti‐apoptotic genes BCL2 and BCL2L1. Genes downregulated by RSK2^Ser227^ inhibition also included those essential for B, cell development, including IKZF1, IKZF3, and PAX5; those in the B cell receptor (BCR) signaling pathway, such as CD19, CD79B, and BLNK; and those involved in APRIL‐mediated/B cell activating factor (BAFF) signaling, such as TNFRSF13B for BAFF and TNFRSF17 for B‐cell maturation antigen (Figure 5B; Table 1). In contrast, canonical pathway analysis of commonly upregulated genes did not identify a major candidate gene underlying anticancer efficacy (Table 2). It remains to be determined if upregulation of the autophagy signal pathway is related to cell protection or cell death in response to inactivation of RSK2^Ser227^ (Table 2). In addition, GSEA analysis based on the GEP results demonstrated that BI‐D1870 treatment caused significant downregulation of several signatures related to tumorigenesis and oncogenic signaling, such as MYC, RAS pathway and NF‐κB pathway, and IL2‐STAT5 pathway which include genes of BCR pathway, such as IKZF2, CD79B, and POU2F1 (Table 3; Figure 5D; Table S5).

**TABLE 1:** Top 30 significantly modulated canonical pathways (*P* < .01) related with commonly downregulated genes by BI‐D1870

	Ingenuity canonical pathways	−log (*P*‐value)	Gene
1	B‐cell receptor signaling	7.51	APBB1IP, BCL2L1, BLNK, CD19, CD79B, CFL1, CREBBP, DAPP1, FCGR2B, GAB1, IKBKE, MEF2C, NFATC1, NFATC2, OCRL, PAX5, PIK3AP1, PIK3C2B, PIK3CG, PTPN6, RASSF5
2	Communication between innate and adaptive immune cells	4.50	CCL3, CCR7, CD79B, CD86, TLR1, TLR10, TLR7, TLR9, TNFRSF13B, TNFRSF17
3	Phospholipase C signaling	4.27	ARHGEF2, ARHGEF3, BLNK, CD79B, CREBBP, FCGR2B, GRAP2, HDAC7, HDAC9, ITGA4, LCK, MEF2C, MEF2D, NFATC1, NFATC2, PLD6, PRKD3, RHOBTB1, RHOH
4	Primary immunodeficiency signaling	4.26	BLNK, CD19, CIITA, LCK, RAG2, TNFRSF13B, UNG
5	Phagosome formation	4.05	FCGR2B, ITGA4, PIK3C2B, PIK3CG, PLCL2, PRKD3, RHOBTB1, RHOH, TLR1, TLR10, TLR7, TLR9
6	PI3K signaling in B lymphocytes	3.89	BLK, BLNK, CD180, CD19, CD79B, DAPP1, FCGR2B, IKBKE, NFATC1, NFATC2, PIK3AP1, PIK3CG, PLCL2
7	TREM1 signaling	3.74	CCL3, CD86, CIITA, FCGR2B, MYD88, TLR1, TLR10, TLR7, TLR9
8	Chronic myeloid leukemia signaling	3.71	BCL2L1, E2F5, E2F8, HDAC7, HDAC9, IKBKE, MYC, PIK3C2B, PIK3CG, SMAD3, TGFBR2
9	Protein kinase A signaling	3.47	AKAP1, AKAP2, CREBBP, DUSP16, DUSP2, DUSP5, NFATC1, NFATC2, PDE4A, PDE4B, PDE8A, PDE9A, PLCL2, PLD6, PPP1R11, PRKD3, PTPN6, PTPN7, PTPN9, PTPRE, PTPRJ, SMAD3, TGFBR2, UBASH3B
10	B‐cell development	3.18	CD19, CD79B, CD86, RAG2, SPN
11	Cardiac hypertrophy signaling (enhanced)	3.16	CD70, CSF2RB, DLG1, HDAC7, HDAC9, IFNLR1, IKBKE, IL21R, IL27RA, ITGA4, MAP3K20, MEF2C, MEF2D, MYC, NFATC1, NFATC2, PDE4A, PDE4B, PDE8A, PDE9A, PIK3C2B, PIK3CG, PLCL2, PLD6, PRKD3, TGFBR2, WNT10A
12	Molecular mechanisms of cancer	3.08	ARHGEF2, ARHGEF3, BCL2, BCL2L1, BIRC3, CASP10, CREBBP, E2F5, E2F8, GAB1, ITGA4, MYC, PIK3C2B, PIK3CG, PRKD3, RASGRP1, RHOBTB1, RHOH, SMAD1, SMAD3, SMAD6, TGFBR2, WNT10A
13	Death receptor signaling	2.96	BCL2, BIRC3, CASP10, DFFB, IKBKE, PARP12, PARP8, PARP9, TNFRSF10A
14	Pancreatic adenocarcinoma signaling	2.90	BCL2, BCL2L1, E2F5, E2F8, HBEGF, PIK3C2B, PIK3CG, PLD6, SMAD3, TGFBR2
15	Altered T cell and B cell signaling in rheumatoid arthritis	2.73	CD79B, CD86, TLR1, TLR10, TLR7, TLR9, TNFRSF13B, TNFRSF17
16	Nur77 signaling in T lymphocytes	2.60	BCL2, CD86, HDAC9, MEF2D, NFATC1, NR4A1
17	Prolactin signaling	2.59	CREBBP, IRF1, MYC, NMI, PIK3C2B, PIK3CG, PRKD3, SOCS2
18	Cell cycle: G1/S checkpoint regulation	2.57	E2F5, E2F8, HDAC7, HDAC9, MYC, PAK1IP1, SMAD3
19	T cell receptor signaling	2.52	GRAP2, IKBKE, LCK, NFATC1, NFATC2, PIK3C2B, PIK3CG, PTPN7, RASGRP1
20	Role of pattern recognition receptors in recognition of bacteria and viruses	2.48	CD70, EIF2AK2, IFIH1, MYD88, OAS1, PIK3C2B, PIK3CG, PRKD3, TLR1, TLR7, TLR9
21	Purine nucleotides de novo biosynthesis II	2.47	ADSS, GART, IMPDH1
22	Role of NFAT in regulation of the immune response	2.40	BLNK, CD79B, CD86, FCGR2B, IKBKE, LCK, MEF2C, MEF2D, NFATC1, NFATC2, PIK3C2B, PIK3CG
23	NF‐κB signaling	2.36	CREBBP, EIF2AK2, LCK, MYD88, PIK3C2B, PIK3CG, TGFBR2, TLR1, TLR10, TLR7, TLR9, TNFRSF17
24	CD28 signaling in T helper cells	2.31	CD86, GRAP2, IKBKE, LCK, NFATC1, NFATC2, PIK3C2B, PIK3CG, PTPN6
25	April‐mediated signaling	2.29	IKBKE, NFATC1, NFATC2, TNFRSF13B, TNFRSF17
26	STAT3 pathway	2.23	BCL2, CSF2RB, IFNLR1, IL21R, IL27RA, MAP3K20, MYC, PTPN6, SOCS2, TGFBR2
27	B‐cell activating factor signaling	2.20	IKBKE, NFATC1, NFATC2, TNFRSF13B, TNFRSF17
28	Th1 and Th2 activation pathway	2.18	BHLHE41, CD86, IKZF1, IL27RA, IRF1, NFATC1, NFATC2, PIK3C2B, PIK3CG, S1PR1, TGFBR2
29	tRNA splicing	2.15	PDE4A, PDE4B, PDE8A, PDE9A, PLD6
30	Systemic lupus erythematosus signaling	2.15	CD72, CD79B, CD86, FCGR2B, LCK, NFATC1, NFATC2, PIK3C2B, PIK3CG, PTPN6, TLR7, TLR9

**TABLE 2:** Significantly modulated canonical pathways (*P* < .01) related with commonly upregulated genes by BI‐D1870

	Ingenuity canonical pathways	−log (*P*‐value)	Molecules
1	DNA methylation and transcriptional repression signaling	3.20	HIST1H4D, HIST1H4K, HIST1H4L, HIST2H4B, HIST4H4
2	Autophagy	3.05	ATG12, ATG4B, CTSK, MAP1LC3B, VPS11, VPS39
3	Phenylethylamine degradation I	2.58	AOC2, AOC3
4	Transcriptional regulatory network in embryonic stem cells	2.27	HIST1H4D, HIST1H4K, HIST1H4L, HIST2H4B, HIST4H4

**TABLE 3:** Significantly downregulated gene sets in BI‐D1870‐treated Jeko‐1 cells (A) and KPUM‐YY1 cells (B) (FDR q‐value < 0.05)

Name	Size	NES	NOM	FDR
			*P*‐value	q‐value
(A) Jeko‐1 cells				
MYC_TARGETS_V2	57	−1.85309	0	0.002687
INFLAMMATORY_RESPONSE	199	−1.8356	0	0.001344
KRAS_SIGNALING_UP	199	−1.78622	0	0.003379
UV_RESPONSE_DN	138	−1.65753	0.001346	0.011184
INTERFERON_GAMMA_RESPONSE	196	−1.60346	0	0.014421
ALLOGRAFT_REJECTION	197	−1.60186	0	0.012174
IL2_STAT5_SIGNALING	196	−1.48374	0.002608	0.045569
(B) KPUM‐YY1 cells				
TNFA_SIGNALING_VIA_NFKB	197	−2.326312	0	0
INTERFERON_GAMMA_RESPONSE	196	−2.1998236	0	0
MYC_TARGETS_V2	57	−2.1888933	0	0
INTERFERON_ALPHA_RESPONSE	94	−2.016676	0	4.25E‐04
INFLAMMATORY_RESPONSE	199	−1.8072267	0	0.002707463
ALLOGRAFT_REJECTION	197	−1.7501364	0	0.004364074
TGF_BETA_SIGNALING	54	−1.7436218	0	0.003914104
IL2_STAT5_SIGNALING	196	−1.7303268	0	0.003582249
UV_RESPONSE_DN	138	−1.7132485	0	0.003184221
G2M_CHECKPOINT	191	−1.4888769	0.002	0.031178242
MYC_TARGETS_V1	197	−1.4880713	0	0.028462738
E2F_TARGETS	197	−1.4487894	0.004123712	0.03797551

Abbreviations: FDR, false discovery rate; NES, normalized enrichment score; NOM, nominal

### 3.5 Normal lymphocytes are not addicted to RSK2^Ser227^ for cell proliferation and survival

**FIGURE 6:**
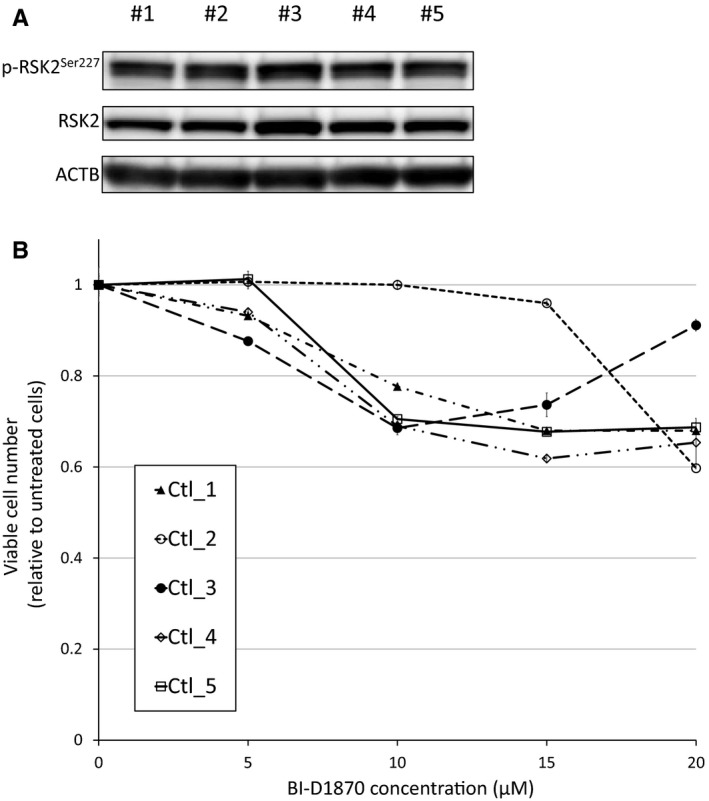
RSK2^Ser227^ status and the effect of RSK2^Ser227^ inhibition in normal lymphocytes. A, RSK2^Ser227^ phosphorylation status in normal lymphocytes from five healthy donors. B, Growth inhibitory effects of BI‐D1870 on normal lymphocytes from five independent healthy donors. Cells were exposed to various concentrations of BI‐D1870 for 48 h, and the relative numbers of viable cells were measured by modified MTT assay

We next examined the activation status of RSK2^Ser227^ in normal lymphocytes. As shown in Figure 6A, RSK2^Ser227^ was activated by phosphorylation in normal lymphocytes from all five healthy donors. However, the exposure to BI‐D1870 up to 20 µmol/L for 48 hours resulted in only modest suppressive effect on normal lymphocytes (Figure 6B), suggesting that normal lymphocytes are less addicted to RSK2^Ser227^ activity for cell proliferation and survival compared with MCL cells.

### 3.6 Strategies for enhancing the antitumor effect of RSK2^Ser227^ inhibition in MCL cells

**FIGURE 7:**
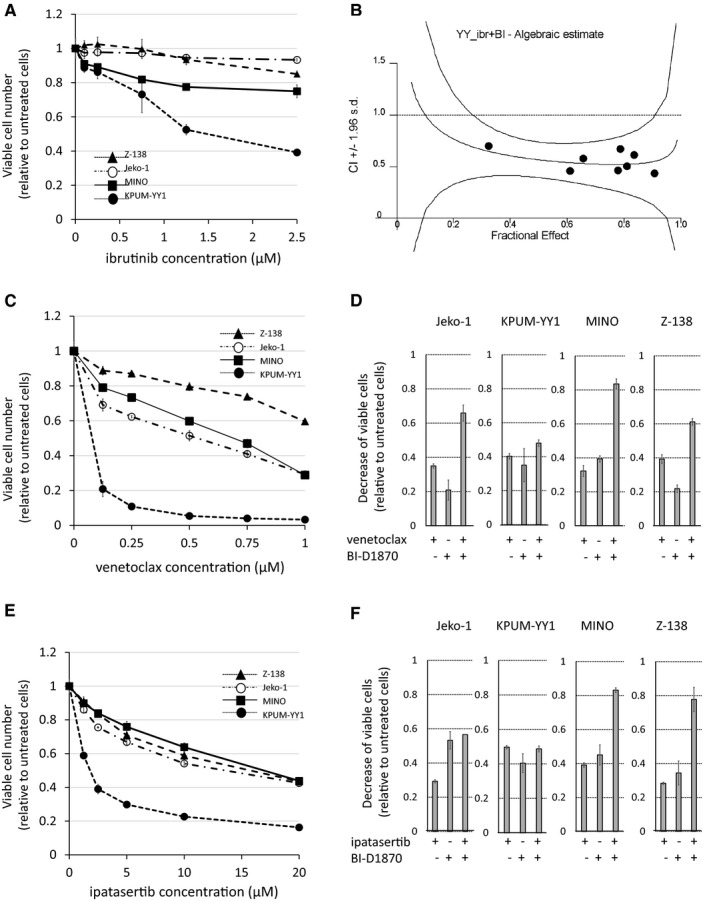
Effects of ibrutinib, venetoclax, and ipatasertib alone and in combination with BI‐D1870 in MCL‐derived cell lines. A,C,E, BI‐D1870 (BI)‐sensitive MCL cells (Jeko‐1, KPUM‐YY1, MINO, and Z‐138 cells) were treated with various concentrations of ibrutinib (ibr) (A), venetoclax (ven) (C), or ipatasertib (ipa) (E) for 48 h and the relative viable cell number was determined by modified MTT assay. Data are shown as the mean ± SD of three independent experiments. B, Combination index (CI) for the combinatory growth inhibitory effect between ibr and BI in KPUM‐YY1 cells. D,F, Growth inhibitory effects of BI (D,F), ven (D), and ipa (F) alone and in combination (D: BI plus ven and F: BI plus ipa) in four BI‐sensitive MCL cell lines. The concentrations used were BI 5.0‐7.5 µmol/L, ven 0.125 µmol/L, and ipa 2.5 µmol/L in Jeko‐1; BI 4.0‐7.5 µmol/L, ven 0.01 µmol/L, and ipa 2.5 µmol/L in KPUM‐YY1; BI 3.0 µmol/L, ven 0.125 µmol/L, and ipa 10.0 µmol/L in MINO; and BI 5.0 µmol/L, ven 0.25 µmol/L, and ipa 10.0 µmol/L in Z‐138. Reductions of viable cell numbers relative to control cells are shown as the mean ± SD of three independent experiments

Finally, we investigated strategies to augment the antitumor effect of BI‐D1870 in MCL‐derived cell lines. As shown in Figure 7A, KPUM‐YY1 cells were found to be only modestly sensitive to ibrutinib within clinically achievable concentration, while Jeko‐1, MINO, and Z‐138 cells were not sensitive to ibrutinib. Therefore, in vitro combinatory growth inhibitory effect of BI‐D1870 and ibrutinib was examined only in KPUM‐YY1 cells, based on the hypothesis that concomitant blockade of RSK2^Ser227^ activity would help augment the modest antitumor effect induced by the blockade of BCR signaling in B cell‐derived neoplastic cells. As a result, the combination of BI‐D1870 and ibrutinib was found to induce synergistic growth inhibitory effect in KPUM‐YY1 cells (Figure 7B). We next examined whether addition of venetoclax enhanced the effect of BI‐D1870, since induction of proapoptotic BH3‐only protein by BI‐D1870 was not significant in the GEP analysis. KPUM‐YY1 cells were highly sensitive to venetoclax, while up to 1 µmol/L venetoclax showed only modest growth inhibitory effects on Jeko‐1, MINO, and Z‐138 cells (Figure 7C). However, venetoclax augmented growth inhibition by BI‐D1870 in Jeko‐1, MINO, and Z‐138 cells, whereas a combinatory effect was not evident in KPUM‐YY1 cells (Figure 7D).

Third, we examined the combinatory effect of BI‐D1870 and ipatasertib because two of the four BI‐D1870‐sensitive cell lines (MINO and Z‐138) exhibited feedback activation of AKT by phosphorylation. Addition of ipatasertib augmented growth inhibition by BI‐D1870 in these cells, but not in Jeko‐1 and KPUM‐YY1 cells, which did not show feedback activation of AKT following BI‐D1870 treatment (Figures 3 and 7E,F).

## 4 DISCUSSION

Deregulated activation of the RAS/RAF/MEK/ERK signaling cascade plays crucial roles in development and progression of solid and hematologic malignancies. All mechanisms for activation of RAS/RAF/MEK/ERK pathway, such as constitutively activating mutations in RAS or RAF, chromosomal abnormalities that transactivate ERK, and cell extrinsic stimuli such as growth factors and cell adhesion, eventually converge upon RSK2, which receives the upstream signal mediated by ERK at its CTKD and then transmit signals to a number of substrates through phosphorylation using its NTKD as the output module. The current study showed that RSK2^Ser227^ in the NTKD is frequently activated by phosphorylation in patient‐derived MCL cells and MCL‐derived cell lines. Moreover, activation of upstream components of the cascade (ERK and/or RSK2‐CTKD) is not always a prerequisite for p‐RSK2^Ser227^ activation in MCL, which is also the case in other mature B cell malignancies, such as MM.^21^, ^22^, ^23^ We have previously shown that constitutive activation of PDPK1 through overexpression activates RSK2‐NTKD in MCL regardless of the activation status of the RAS/MAPK/ERK pathway,^24^ but a mutation that activates the RAS/RAF/MEK/ERK pathway directly is rare in MCL.^26^, ^27^, ^28^, ^29^ These results provide important information showing that activation of RSK2^Ser227^ is critical in cancer pathogenesis, irrespective of the existence of an activating chromosomal abnormality or mutation in the RAS/MAPK/ERK pathway. Moreover, this study showed less suppressive effect of BI‐D1870, a specific inhibitor for RSK2^Ser227^, on the survival of normal lymphocytes, despite the active status of RSK2^Ser227^. These suggest that normal lymphocytes are less addicted to RSK2 activity compared with MCL cells, and we expect that the difference in the dependency to RSK^Ser227^ between MCL and normal lymphocyte may produce the chance for targeted treatment against RSK2^Ser227^. Because cells completely lacking RSK2^Ser227^ activity were rarely offered, it was difficult to completely exclude off‐target effect or nonspecific toxicity of BI‐D1870 in this study. Nevertheless, these collectively suggest that RSK2^Ser227^ and the NTKD are potential therapeutic targets in many types of cancers, including MCL regardless of mutation status of TP53 which associates with adverse prognosis.^15^, ^16^, ^17^, ^26^, ^27^, ^28^, ^29^

In this study, we showed that BI‐D1870 inhibition of p‐RSK2^Ser227^ had an antiproliferative effect via G_2_/M cell cycle blockade and induction of apoptosis. Also, RSK2 gene knockdown inhibited cell proliferation of MCL‐derived cell lines. It was conceivable that MCL cells that are more dependent to ERK/RSK2 pathway could be more sensitive to the blockade of RSK2^Ser227^. Indeed, MINO cells and Z‐138 cells with low basal AKT activity were highly sensitive to BI‐D1870, even though the rebound AKT phosphorylation was observed following RSK2^Ser227^ inhibition. In addition, MINO cells and Z‐138 cells were not highly sensitive to ipatasertib. These suggested that MINO cells and Z‐138 cells may be more addicted to ERK/RSK2 pathway, but not to AKT signaling, compared with Jeko‐1 cells and KPUM‐YY1 cells. In contrast, Jeko‐1 cells and KPUM‐YY1 cells with high basal AKT activity may use both ERK/RSK2 and AKT signaling pathways for their cell survival, and, therefore, this could at least partly underly the slightly less sensitivity to BI‐D1870, while the higher sensitivity to ipatasertib compared with MINO cells and Z‐138 cells. To further clarify the molecular mechanism underlying the anticancer effect of RSK2^Ser227^ inhibition in MCL, we conducted a GEP with signal pathway analysis to identify target genes/pathways of RSK2^Ser227^. BI‐D1870 treatment, rather than RSK2 gene knockdown, was selected for inhibition of RSK2‐NTKD activity in this analysis because we wanted to investigate gene expression changes directly induced by inhibition of the kinase activity of RSK2‐NTKD. We found a large concordance of gene expression changes due to BI‐D1870 in KPUM‐YY1 and Jeko‐1 cells, and inhibition of RSK2 kinase activity downregulated a series of genes involved in development, activation, survival, and oncogenesis in B cells. Such downregulation of oncogenes (c‐MYC and MYB) and genes involved in BCR (BLNK, CD19, and CD79B), PI3K pathways (PIK3AP1 and PIK3CG), and B cell activating factor signaling (TNFRSF17 and TNFRSF13B) may be associated with the antiproliferative effect of BI‐D1870.^34^ Downregulation of antiapoptotic genes, such as BCL2 and BCL2L1, is likely to be associated with induction of apoptosis. In addition, the assessments with GSEA revealed that the blockade of RSK2^Ser227^ potently interferes with interferon γ signaling and IL‐2 signaling have been reported to be involved in molecular pathophysiology of MCL.^27^ Thus, these results show that blockade of RSK2‐NTKD enables concomitant downregulation of genes that cooperatively promote B cell tumorigenesis, especially MCL.

Finally, we searched for a potential strategy to augment the effect of p‐RSK2^Ser227^ targeting in anti‐MCL therapy. Clinical effects of ibrutinib and venetoclax in MCL have been shown,^35^, ^36^ but there is an unmet need for a strategy that can enhance these effects. First, we found that a combination of BI‐D1870 and ibrutinib resulted in significantly greater growth inhibition compared with that induced by either agent alone in ibrutinib modestly sensitive KPUM‐YY1 cells. Given the GEP results, we speculate that overlapping blockade of the BCR signaling pathway and reduction of antiapoptotic BCL2 underlie the combinatory antiproliferative effects in MCL cells harboring modest sensitivity to ibrutinib. Also, as shown in ibrutinib‐insensitive MCL cell lines, blockade of RSK2^Ser227^ activity is likely to constitute salvage strategy for ibrutinib‐resistant MCL.

Next, we investigated whether venetoclax enhances the antiproliferative effect of BI‐D1870. The degree of dependence on BCL2 and other anti‐apoptotic BCL2 family proteins varies among cells and tissue types, and indeed, KPUM‐YY1 cells were highly sensitive to venetoclax, a BCL2‐specific inhibitor, while the other three cell lines only showed moderate sensitivities to venetoclax. Intriguingly, an additional antiproliferative effect of venetoclax combined with BI‐D1870 was observed in these three cell lines, but not in the highly venetoclax‐sensitive KPUM‐YY1 cells. These results suggest that in BCL2‐addicted tumor cells, venetoclax monotherapy inhibits proliferation, but not necessarily when combined with BI‐D1870, which reduces other anti‐apoptotic proteins, such as BCL2L1, in these cells. In BCL2‐nonaddicted tumor cells, in contrast, blockade of BCL2 by venetoclax increases susceptibility to the antitumor effect of BI‐D1870.

Third, we found that a combination of BI‐D1870 and ipatasertib significantly enhanced growth inhibition of MCL‐derived cells that exhibited feedback activation of AKT following BI‐D1870 treatment (MINO and Z‐138 cells), while this effect was not observed in cells that did not show feedback AKT activation subsequent to RSK2^Ser227^ inhibition. AKT inhibitors are promising new antitumor agents.^37^, ^38^ The results of this study indicate that AKT reactivation causes refractoriness to an anticancer effect induced by the inhibition of RSK^Ser227^ in a cell context‐dependent manner. The mechanism underlying AKT reactivation following RSK2^Ser227^ inactivation is unknown.

In conclusion, our results suggest that RSK2^Ser227^ in the NTKD is a potential therapeutic target in MCL. Future work is needed to develop a bioavailable RSK2 NTKD‐specific inhibitor.

## CONFLICT OF INTEREST

JK has received research funding from Celgene, Kyowa Kirin, Chugai Pharmaceutical, Ono Pharmaceutical, Sanofi, Eisai, Bristol‐Myers Squibb, Sysmex, Astellas Pharma, Pfizer, Sumitomo Dainippon Pharma, Nippon Shinyaku, MSD, Fujimoto Pharmaceutical and Otsuka Pharmaceutical; has received honoraria from Janssen Pharmaceutical KK, Celgene Corporation, Kyowa Kirin, Chugai Pharmaceutical, Ono Pharmaceutical, Sanofi, Eisai, Bristol‐Myers Squibb, Astellas Pharma, Pfizer, Nippon Shinyaku, Sumitomo Dainippon Pharma, Fujimoto Pharmaceutical, Abbvie, and Otsuka Pharmaceutical; and is a consultant for Janssen Pharmaceutical KK, Celgene, Bristol‐Myers Squibb, Sanofi, and Abbvie. MT has received research funding from Kyowa Kirin, Chugai Pharmaceutical, Eisai, Bristol‐Myers Squibb, and Astellas Pharma. TK has received research funding from MSD, and has received honoraria from Chugai Pharmaceutical, Ono Pharmaceutical, Eisai, and Nippon Shinyaku. TT has received research funding from Nippon Shinyaku.

## AUTHOR CONTRIBUTION

YMK, TT, YC, KT, SKO, YF, DN, RI, JY, YKK, TK, and SH performed experiments. YMK and JK analyzed the data. YMK and JK drafted the manuscript. JK and MT designed experiments and the overall study. MT and JK supervised the study.

## Supporting information

Table S1

Table S2

Table S3

Table S4

Table S5

Supplementary Material
