# Supplementary material for: Serine‐227 in the N‐terminal kinase domain of RSK2 is a potential therapeutic target for mantle cell lymphoma
Source: Cancer Med. 2020 May 18;9(14):5185–99. doi: 10.1002/cam4.3136 (PMC7367644; doi:10.1002/cam4.3136)
Supplement: Supplementary file 1 — Table S1 [file CAM4-9-5185-s001.docx]

**Supplementary Table 1. Clinical information and cytogenetic data of five patients with mantle cell lymphoma subjected to phosphorylated RSK2^Ser227^ immunohistochemical (IHC) staining.**

| **Pt. No.**  **/Tissue** | **Age**  **/Sex** | **IHC staining pattern** | **G-banding** | **FISH**  **(IgH/CCND1)** | **CS** | **MIPI** |
| --- | --- | --- | --- | --- | --- | --- |
| 1  /spleen | 74/M | CD5(+), CD10(-), CD20(+), CD79a(+), BCL2(+), CCND1(+), | 45, XY, add(1), (p11), -8, -11, t(11;14)(q13,q32), -13, add(15)(q22), -16, +mar1, +mar2, +mar3 [2/6] and other 4 cell with 45 chromosomes | No data | Ⅳ | Int |
| 2  /Tonsil | 59/M | CD5(+), CD10(-), CD20(+), CD79a(+), BCL2(+), CCND1(+) | Not available | 49% | Ⅳ | Low |
| 3  /lymph node | 66/M | CD5(+), CD10(+), CD20(+), CD79a(+), BCL-2(+), CCND1(+), BCL6(-), MUM1(-) | Not available | 95% | Ⅳ | Int |
| 4  /lymph node | 46/M | CD5(+), CD10(-), CD20(+), CD79a(+), CCND1(+) | 50, XY, der(1)(p36.1)add(1)(q32), +7, add(8)(p11.2), add(9)(p11)x2, -10, -11, -11, +12,add(12)(q24.1)x2, add(13)(q32)x2, der(14)? t(11;14)(q13;q32), add(15)(q11.2), +18, add(20)(q11.2), +der(?) t (?;11)(?;q13), +mar1, +mar2, +mar3 [11/20]/46, XY[1/20], and other 1cell with 49 chromosome, 5 cells with 50 chromosomes, and 2 cells with 51 chromosomes | 49% | Ⅲ | Low |
| 5  /lymph node | 73/M | CD5(+), CD10(-), CD20(+), CD79a(+), CCND1(+), BCL6(-) | 77<2n>, -X, -Y, +1, +2, +3, add(4)(q11), -5, +7, +8, add(11)(p11.2), -12, -12, -13, -13, -14, -15, -18, +19, +21, +34mar [1/13]/46, XY [12/13] | No data | Ⅳ | Int |

BM; Bone marrow, CCND1; cyclin D1, CS: Clinical Stage, FISH; fluorescence in situ hybridization, Int; intermediate, M; male, MIPI; The Mantle Cell Lymphoma International Prognostic Index, Pt.No.; Patient number
