# Supplementary material for: Serine‐227 in the N‐terminal kinase domain of RSK2 is a potential therapeutic target for mantle cell lymphoma
Source: Cancer Med. 2020 May 18;9(14):5185–99. doi: 10.1002/cam4.3136 (PMC7367644; doi:10.1002/cam4.3136)
Supplement: Supplementary file 2 — Table S2 [file CAM4-9-5185-s002.docx]

| **Supplementary Table 2. Chromosomal features of MCL-derived cell lines.** | |
| --- | --- |
| **Cell line** | **Karyotype by metaphase spreads** |
| Jeko-1 | 4n; 71, XX, t(X;9)(p11.2;q34.1), der(X)t(X;9)(p11.2;q34.1), +der(X)t(X;9)(p11.2;q34.1), der(1)t(1;2)(p22.1;q11.2)x2, del(2)(q11.2)x2, der(3)(3pter→3q12::8q24→8q24::3q12→3q29::7q32→7qter), -3, -4, der(5)t(5;12)(p15.1; q24.1)x2, -5, der(6)(6qter→6p21.3::8q24→8q24::?)x2, -6, -6, der(7)t(4;7)(q27;q31), -8, -8, der(9)t(X;9)(q22;q22), der(9)t(9;14)(q22;q22), der(9) (7pter→7p15::9q34.1→9p13::12q::1p32→1pter), -9, der(10)t(3;10)(p21;p11.2)x2, der(11)t(11 ;14)(q13;q32), del(12)(q11), -12, der(13)t(1;13)(p36.1;q32)x2, der(13)t(1;13)(q32;p11.2)x2, +der(13)t(13;16)(p11.2;q22), i(14q),der(14)(17qter→17q11.2::14p11.2→14q32::11q13→11qter), -14, -14, -15, del(16)(q11.1), -16, -17, -17, -18, -18, -19, -19, -20, der(21)t(16;21)(p11.2; p11.2)x2, -21, -21, der(22)t(21;22)(q11.2;q11.2)x2 (m07) |
| JVM-2 | 46, XX, t(4;13)(q31.3;q14.1), der(8)t(3;8)(q13.2;p21.1), t(11;14)(q13;q32) (m01, 02, 03, 04, 05) |
| KPUM-YY1 | 50, XY, +7, t(8;14)(q24;q32), der(11)t(11;14)(q13;q32), +dup(12)(q?q?), +13, der(14)(14pter→14q32::11q13→11q?), dic(1;15)(q21;p11.2), i(17q), ?del(18)(q21)x2, +18 (m03, 04)  50, XY, der(6)t(6;17)(q23;q11.2), +7, t(8;14)(q24;q32), der(11)t(11;14)(q13;q32), +dup(12)(q?q?), +13, der(14)(14pter→14q32::11q13→11q?), dic (1;15)(q21;p11.2), ?del(18)(q21)x2, +18 (m05)  50, XY, +7, t(8;14)(q24;q32), der(11)t(11;14)(q13;q32), +dup(12)(q?q?), +13, der(14)(14pter→14q32::11q13→11q?), der(16)t(16;20)(q24;q11), ?del (18)(q21)x2, +18 (m02) |
| MINO | 4n-; 75, XXYY, der(1)t(1;9)(q44;q12), -1, del(3)(p25)(q27), -3, -3, -4, -5, i(6p), -6, +7, t(8;14;11)(q24;q32;q13)x2, -8, -9, -9, -10, der(11)(11pter→11q13::14q24→14q32::8q24→8qter), -11, -12, der(13)t(3;13)(q25;q32), -13, -14, -15, -16, -17, der(18)(18pter→18q22::18q21.1→18q21.3::3q21→3qter), -18, -19, -22 (m01, 03) |
| Z-138 | 4n-; 83, XXYY, +1, t(2;5)(q21;q15)x2, del(3)(p12)x2, der(3)(7qter→7q22 :: 3p21→3q21::?::7q11.2→7qter)x2, t(4;14)(q12;q32.1), -4, +del(5)(q11.2q15), +del(5)(q11.2), der(6)t(6;8)(p25;q24)x2, -6, -6, del(7)(q31), der(7)t(7;?;7)(7qter→7q11.2::?::7q22→7qter)x2, -7, t(8;14;11)(q24;q32;q13), der(8)t(8;14;11), -9, -9, -9, -10, -10, der(11)t(8;14;11), der(11)t(9;11)(q21;p11.2), der(12)t(8;12)(q24;q15)x2, der(12)t(11;12)(q21;p11.2), -12, der(14)t(4;14), -14, der(15)t(6;15)(p11.2;p11.2)x2, -17, der(18)t(12;18)(p11.1;p11.1)x2, -18, der(19)(19pter→19q13.1::14q24→14q32::8q24→8qter)x2, -19, +20, +22 (m01) |
