# Supplementary material for: Serine‐227 in the N‐terminal kinase domain of RSK2 is a potential therapeutic target for mantle cell lymphoma
Source: Cancer Med. 2020 May 18;9(14):5185–99. doi: 10.1002/cam4.3136 (PMC7367644; doi:10.1002/cam4.3136)
Supplement: Supplementary file 3 — Table S3 [file CAM4-9-5185-s003.docx]

**Supplementary Table 3. Primers utilized in quantitative RT-PCR.**

| **Gene** | **Forward (5’-3’)** | **Reverse (3’-5’)** |
| --- | --- | --- |
| *ACTB* | TCT ACA ATG AGC TGC GTG TG | TGG ATA GCA ACG TAC ATG GC |
| *BCL2* | TGG ATG ACT GAG TAC CTG AAC C | AAT CAA ACA GAG GCC GCA TG |
| *BCL2L1* | ATG CAG GTA TTG GTG AGT CG | CTG CTG CAT TGT TCC CAT AG |
| *BLNK* | AAA GCA CCT CCA AGT GTT CC | CCG AGT GCT CAT CTG GAT TTT C |
| *CD79B* | TGG ACA AGG ATG ACA GCA AG | ACC ACT TCA CTT CCC CTG TC |
| *c-MYC* | TCG GAT TCT CTG CTC TCC TC | TCC TCA TCT TCT TGT TCC TCC TC |
| *c-MYB* | ATC GAA CAG ATG TGC AGT GC | CCA ACG TTT CGG ACC GTA TTT C |
| *IKZF1* | ATG CTG ATG AGG GTC AAG AC | ACG CCC ATT CTC TTC ATC AC |
| *PAX5* | TTC CAG TCA CAG CAT AGT GTC C | CTC TTG CGC TTG TTG GTG TC |
| *RSK2* | CGC TGA GAA TGG ACA GCA AAT | TCC AAA TGA TCC CTG CCC TAA T |
| *TNFRSF17* | GCT CTT GCT GCA TTT GCT CTG | GAC AAG AAT GGT TGC GCC TTC |
