# Supplementary material for: Serine‐227 in the N‐terminal kinase domain of RSK2 is a potential therapeutic target for mantle cell lymphoma
Source: Cancer Med. 2020 May 18;9(14):5185–99. doi: 10.1002/cam4.3136 (PMC7367644; doi:10.1002/cam4.3136)
Supplement: Supplementary file 6 — Supplementary Material [file CAM4-9-5185-s006.docx]

**Supplementary Legend**

**Supplementary Table 1.** Clinical information and cytogenetic data for five patients with mantle cell lymphoma subjected to phosphorylated RSK2^Ser227^ immunohistochemical (IHC) staining.

**Supplementary Table 2.** Chromosomal features of MCL-derived cell lines utilized in this study.

**Supplementary Table 3**. Primers utilized in quantitative RT-PCR.

**Supplementary Table 4**. Results of gene expression profiles for Jeko-1 and KPUM-YY1 cells treated by BI-D1870.

**Supplementary Table 5.** Genes significantly modulated by BI-D1870 treatment those are involved in interleukin-2-STAT5 signaling pathway in MCL cell lines
